# Validation of the imperial psychedelic predictor scale

**DOI:** 10.1017/S0033291724002204

**Published:** 2024-09

**Authors:** Michael Angyus, Sarah Osborn, Eline Haijen, David Erritzoe, Joseph Peill, Taylor Lyons, Hannes Kettner, Robin Carhart-Harris

**Affiliations:** 1 Department of Brain Sciences, Centre for Psychedelic Research, Imperial College London, London, UK; 2 Department of Neuropsychology and Psychopharmacology, Faculty of Psychology and Neuroscience, Maastricht University, Maastricht, the Netherlands; 3 Departments of Neurology and Psychiatry, University of California San Francisco, San Francisco, CA, USA

**Keywords:** pharmaceuticals, predicting psychedelic response, psychedelic preparation, psychedelics, psychometrics, safety, set and setting

## Abstract

**Background:**

Access to psychedelic drugs is liberalizing, yet responses are highly unpredictable. It is therefore imperative that we improve our ability to predict the nature of the acute psychedelic experience to improve safety and optimize potential therapeutic outcomes. This study sought to validate the ‘Imperial Psychedelic Predictor Scale’ (IPPS), a short, widely applicable, prospective measure intended to be predictive of salient dimensions of the psychedelic experience.

**Methods:**

Using four independent datasets in which the IPPS was completed prospectively – two online surveys of ‘naturalistic’ use (*N* = 741, *N* = 836) and two controlled administration datasets (*N* = 30, *N* = 28) – we conducted factor analysis, regression, and correlation analyses to assess the construct, predictive, and convergent validity of the IPPS.

**Results:**

Our approach produced a 9-item scale with good internal consistency (Cronbach's *α* = 0.8) containing three factors: set, rapport, and intention. The IPPS was significantly predictive of ‘mystical’, ‘challenging’, and ‘emotional breakthrough’ experiences. In a controlled administration dataset (*N* = 28), multiple regression found set and rapport explaining 40% of variance in mystical experience, and simple regression found set explained 16% of variance in challenging experience. In another (*N* = 30), rapport was related to emotional breakthrough explaining 9% of variance.

**Conclusions:**

Together, these data suggest that the IPPS is predictive of relevant acute features of the psychedelic experience in a broad range of contexts. We hope that this brief 9-item scale will be widely adopted for improved knowledge of psychedelic preparedness in controlled settings and beyond.

## Introduction

Psychedelics are receiving widespread attention as clinical trials show positive results with psychedelic-therapy for a wide range of psychiatric disorders – for recent meta-analysis see Zeifman et al. (2022). Legal access to psychedelic-therapy is available in Australia and the US state of Oregon, and FDA approval for the first psychedelic intervention is also under review. In this context of liberalizing access, it is imperative that we develop our ability to predict the nature of psychedelic experiences – so as to mitigate risk and potentially maximize benefit.

Qualities of the subjective experience have been found to be predictive of subsequent positive and negative therapeutic outcomes (Herrmann et al., 2022; Roseman, Nutt, & Carhart-Harris, 2018), indicating the nature of experience may be an important determinant of subsequent therapeutic trajectories (Yaden & Griffiths, 2020). Specifically, so-called ‘peak’ or ‘mystical-type experiences’ (Barrett, Johnson, & Griffiths, 2015; Bogenschutz et al., 2015; Garcia-Romeu, Griffiths, & Johnson, 2015; Johnson, Garcia-Romeu, Cosimano, & Griffiths, 2014; Roseman et al., 2018; Studerus, Gamma, & Vollenweider, 2010), ‘challenging experiences’ (Barrett, Bradstreet, Leoutsakos, Johnson, & Griffiths, 2016; Gashi, Sandberg, & Pedersen, 2021; Roseman et al., 2018), and ‘emotional breakthrough’ (Dougherty et al., 2023; Haijen et al., 2018; Kettner et al., 2021; Lyons et al., *in prep*; Murphy et al., 2021; Peill et al., 2022; Roseman et al., 2019) have been related to therapeutic outcomes (Peill et al., 2022).

While recognized for its importance, the nature of the acute subjective experience induced by psychedelics has been notoriously difficult to predict (Aday, Davis, Mitzkovitz, Bloesch, & Davoli, 2021). Researchers have long speculated that ‘set’ and ‘setting’ (Leary et al., 1965), and additionally the psychosocial ‘matrix’ (Eisner, 1997) as important modulators of psychedelic response, and the field has generally settled on certain guidelines that are intended to support safe and positive experiences (Johnson, Richards, & Griffiths, 2008). However, recent studies are beginning to advance on these to propose and test the contributions of specific elements to the nature and therapeutic success of psychedelic experiences (e.g. Heinzerling et al., 2023).

**Figure 1.**
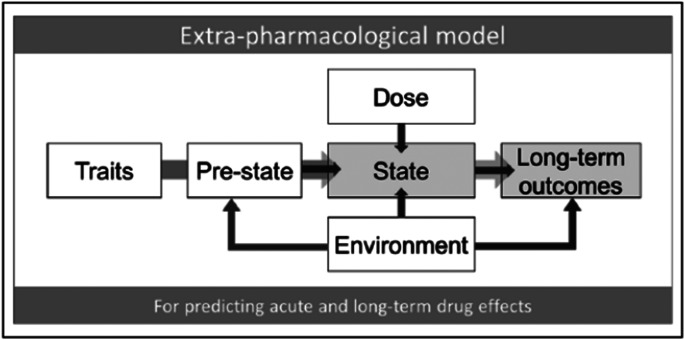
Predictors of psychedelic outcomes (from Carhart-Harris et al., 2018) in which our operational definition bears most relevance to the ‘pre-state’ component.

A range of predictors are being investigated, including environment (Kaelen et al., 2018; Kettner et al., 2021), dosage (Barsuglia et al., 2018; Bremler, Katati, Shergill, Erritzoe, & Carhart-Harris, 2023; Griffiths et al., 2011; Holze et al., 2021; Kangaslampi, Hausen, & Rauteenmaa, 2020; Lyvers & Meester, 2012; Madsen et al., 2019), traits (Russ, Carhart-Harris, Maruyama, & Elliott, 2019a; Russ, Carhart-Harris, Maruyama, & Elliott, 2019b; Smigielski, Scheidegger, Kometer, & Vollenweider, 2019; Studerus, Gamma, Kometer, & Vollenweider, 2012), and ‘pre-state’ (see Fig. 1 for visual representation). We operationally define ‘pre-state’ as including the following elements: (1) expectations, (2) intentions, (3) present mood and mental state, and (4) present inter-personal feelings toward those in their presence (Carhart-Harris et al., 2018). With ‘pre-state’, we sought to capture aspects of ‘set’ (and aspects of ‘setting’ as they translate into ‘set’) that could be assessed shortly before taking a psychedelic.

Psychological preparation is hypothesized to be important for response to psychedelics – as implied here (Haijen et al., 2018), but approaches toward promoting this vary greatly across clinical trials (Thal et al., 2022). A scale that assesses state-level variables that are relevant to ‘pre-state’, such as ‘preparedness’, could aid in the development of preparatory interventions, bolstered by an evidence base that is presently lacking. To our knowledge, there have been four modern research studies assessing our operationally defined ‘pre-state’ (Haijen et al., 2018; McAlpine, Blackburne, & Kamboj, 2024; Russ et al., 2019a, 2019b).

Russ et al. (2019a, 2019b) developed a two-factor inventory intended to assess the constructs of ‘surrender’ and ‘preoccupation’, finding that *preoccupation* was correlated with adverse reactions, while *surrender* was correlated with ‘ego dissolution’ and *mystical-type experiences*. McAlpine et al. (2024) developed a 20-item scale of *preparedness*. When prospectively testing their proposed scale on a psilocybin retreat dataset (*N* = 46), they found that a group with high *preparedness* scores showed significantly better responses to ‘Depression Anxiety and Stress Scale’ (DASS-21) scored *depression*, *anxiety*, and *stress*. Haijen et al. (2018) conducted a rigorous prospective survey design with questionnaires administered 1 week prior, 1 day before, 1 day after, 2 weeks after, and 4 weeks following the day a participant planned to use a psychedelic. Using a self-constructed scale aimed at ‘pre-state’, this study found that ‘pre-state’ was positively correlated with *mystical-type experiences* and *well-being* improvements and negatively correlated with acute *challenging experience*.

The present study intends to refine and formally validate Haijen et al.'s scale, calling it the ‘Imperial Psychedelic Predictor Scale’ (IPPS). Specifically, we used extended data from Haijen's survey (*N* = 741) (Haijen et al., 2018), a study of ceremony usage of psychedelics (*N* = 836) (Kettner et al., 2021), and two controlled trials (*N* = 30 and *N* = 28) (Carhart-Harris et al., 2021; Lyons et al., *in prep*) to assess the internal, convergent, and predictive validity of the IPPS. In the process of validation, the scale was reduced to items that reliably predict acute experience across independent datasets.

## Method

### Data collection

The data used in this study were collated from four studies approved by the Joint Research Compliance Office and the Imperial College Research Ethics Committee. Written consent was obtained prior to admission to the studies. Due to the observational nature of two source studies, all psychedelic drugs were taken by the individuals own accord, without any experimental control. Data from controlled research received favorable opinion from National Research Ethics Service London-West London, was sponsored and approved by Imperial College London's Joint Research and Complication Organisation, adopted by the National Institute of Health Research Clinical Research Network and reviewed and approved by the Medicines and Healthcare products Regulatory Agency. Storage and dispensing licenses for schedule 1 drugs were obtained to enable safe formulation by Guys and St Thomas’ Hospital Pharmacy. Psilocybin for the controlled trials was provided by COMPASS Pathways.

### Psychological measures

Measures relevant to the present study are outlined below.

### EBI

The ‘Emotional Breakthrough Inventory’ (EBI) is used to assess *emotional breakthrough* via a visual analogue scale from 0 to 100. Internal consistency of EBI is excellent (Cronbach's *α* = 0.932). Predictive validity of EBI was first demonstrated through correlation with *well-being* 2-weeks post psychedelic experience (*r* = 0.294, *p* = 0.005; Roseman et al., 2019) and has since been further supported (Kettner et al., 2021; Murphy et al., 2021; Peill et al., 2022).

### MEQ

The ‘Mystical Experience Questionnaire’ (MEQ-30) (Maclean, Leoutsakos, Johnson, & Griffiths, 2012), used here, is a revised and shorter version of an earlier measure developed by Pahnke and Richards (1966). MEQ has excellent internal consistency (Cronbach's *α* = 0.933) and strong predictive validity with respect to measures associated with long-term *well-being* (Garcia-Romeu et al., 2019; Griffiths, Richards, Johnson, McCann, & Jesse, 2008).

### CEQ

The ‘Challenging Experience Questionnaire’ (CEQ) pulls from other psychedelic questionnaires (Griffiths, Richards, McCann, & Jesse, 2006; Strassman, Qualls, Uhlenhuth, & Kellner, 1994; Studerus et al., 2010) to assess distress during a psychedelic experience. Each of the 26 questions of the CEQ are measured on a 5-point Likert scale and relate to one of the seven dimensions: ‘fear’, ‘paranoia’, ‘insanity’, ‘physical distress’, ‘isolation’, ‘death’, and ‘grief’. Internal consistency of the subscales ranges from fair to very good (Cronbach's *α* = 0.65–0.89). Internal validity of the scale as whole has been reported as excellent (Cronbach's *α* = 0.95; Davis et al., 2021).

### Communitas

The Communitas Scale, introduced by Kettner et al. (2021), assesses perceived togetherness and shared humanity (*communitas*) – a social dimension of psychedelic experience – through eight questions. The Communitas Scale shows excellent internal consistency (*α* = 0.92). Concurrent validity is shown through correlation with psychological *well-being* (*r* = 0.22) and path analyses indicating a mediating effect on long-term outcomes.

### Surrender

The *surrender* scale was introduced by Russ et al. (2019a) to assess the ‘set’ of a psychedelic user before dosing. The ten items that constitute the *surrender* factor showed high internal consistency (*α* = 0.918), and the four items that constitute *preoccupation* showed good internal consistency (*α* = 0.757) and were demonstrated in two different datasets to be correlated and predictive of psychedelic experience (Russ et al., 2019a, 2019b). This study used a reduced, five-item version of this scale administered before dosing sessions in Haijen et al. (2018).

### IPPS construction

Twelve items were originally used in Haijen et al. (2018) and were employed here in the development phase of the IPPS. These were created by senior author, RC-H, based on prior knowledge of psychedelic phenomenology and literature, conversation with colleagues at the Centre for Psychedelic Research, Imperial College London, and direct work within the field of psychedelic science and medicine. The items were devised based on observations of responses to psychedelics seen first-hand (e.g. in research studies and trials), principles implied by quantitative research findings, and a familiarity with the human research literature on classic serotonergic psychedelics plus a broader reading of psychological literature. The idea was to capture aspects of an individual's ‘pre-state’ related to (1) their preparedness for the experience, (2) their readiness to relinquish top-down psychological control and let go to the experience, (3) their feelings toward others with them for the experience (e.g. their therapists, facilitators, or guides), and (4) their intentions for having the experience.

### Drug type and dose

Two datasets used observational data with a range of drug type and dose. Participants had the following options to indicate which psychedelic they had taken. Dose varied from the equivalent of 50–300 μg of lysergic acid diethylamide (LSD). The controlled datasets are from two trials in which 25 mg of psilocybin was administered.

### Software

A combination of R version 4.2.2 and MATLAB version R2021b was used to complete the following analyses. Additional packages used in R include: *dyplr*, *stats*, and *psych*. R was used to complete principal component analysis (PCA) and regression analyses. MATLAB was used to complete correlation matrices and regression analyses.

### Factor analysis

IPPS scores from all 12 questions were included in an exploratory factor analysis by PCA. The internal consistency of resulting factors was assessed with Cronbach's alpha. PCA indicated three factors: *set*, *rapport*, and *intention*.

### Scale reduction

Two 12 × 6 correlation matrices were run testing the original 12 items of the IPPS with 6 outcomes of interest, namely, MEQ, CEQ, and EBI. Items were retained if they showed significant correlations with outcomes in both of the independent datasets (see online Supplementary materials).

### Validation

#### Predictive validity

To compare the predictive validity of the reduced IPPS across different datasets, individual factors and total IPPS scores were related to outcomes of interest in recreational use (*cohort*), ceremony use (*ceremony*), and two sets of controlled data (*depression trial*, *healthy volunteer*). The *depression trial* involved two dosing sessions, with the IPPS administered just before each ingestion of psilocybin. We therefore treated each dosing session separately (*N* = 30). The *healthy volunteer* study involved a single dosing session with IPPS again administered prospectively (*N* = 28).

Three multiple regressions were completed with acute measures (CEQ, MEQ, and EBI) as the dependent variable. Subfactors of IPPS were set as independent variables. Each model was trained on *cohort* data and tested on concatenated *ceremony* and *depression trial* datasets (*healthy volunteer* was not used in this analysis due to item differences explained below).

After this train–test regression analysis, simple regression fits were conducted on the two controlled datasets to further assess the predictive validity of the IPPS. An incomplete version of the IPPS was used in the *healthy volunteer* dataset due to its premature state at the start of the study. The version used maintained the *rapport* factor, but not *intention* and with a modified *set.* The modified *set* omitted items two and three of the final scale (Table 1).

**Table 1.** Final IPPS

Item	Set	Rapport	Intention
1. I feel ready to surrender to whatever will be	**0.512**	0.187	0.114
2. I feel open to the upcoming experience	**0.717**	0.163	0.194
3. I feel well prepared for the upcoming experience	**0.585**	0.154	0.334
4. I feel comfortable about the upcoming experience	**0.734**	0.110	0.132
5. I am in a good mood	**0.560**	0.233	
6. I feel anxious	**−0.558**		0.323
7. I have a clear intention for the upcoming experience	0.203		**0.459**
8. I have a good feeling about my relationship with the group/people who will be with me during my experience^a^	0.165	**0.899**	−0.103
9. I have a good relationship with the main person/people who will look after me during the upcoming experience	0.125	**0.672**	

This table displays final 9-item IPPS with factor loadings. Bold numbers indicate which factors the items belong to. Items 1–6 fall under *set*, item 7 *intention*, and items 8 and 9 *rapport*.

a This item is not included in one-on-one dosing settings.

#### Convergent validity

In light of our aim to predict positive outcomes based on *set*, the *surrender* scale was used to convergently validate the IPPS. This was done through correlations between total IPPS scores as well as IPPS factor scores.

## Results

### Demographics

Demographics for *cohort* (*N* = 741), *ceremony* (*N* = 836), *depression trial* (*N* = 30), and *healthy volunteer* (*N* = 28) datasets are displayed in Table 2.

**Table 2.** Demographics

		Cohort	Ceremony	Depression trial	Healthy volunteer
*Demographic responses*	Answered	654 (88.26%)	779 (93.18%)	30 (100%)	28 (100%)
	Blank	87 (11.74%)	57 (6.82%)	0 (0%)	0 (0%)
*Gender*	Male	485 (74.16%)	419 (53.79%)	19 (63.33%)	16 (57.14%)
	Female	165 (25.23%)	357 (45.83%)	11 (36.67%)	12 (42.86%)
	Other	4 (0.61%)	3 (0.39%)	0 (0%)	0 (0%)
*Age*	Mean ± s.d.	28.9 ± 10.45	45.9 ± 12.79	43.3 ± 11.73	40.6 ± 8.65
*Education level*	Left school before age 16 without qualifications	8 (1.22%)	4 (0.51%)	4 (13.33%)	0 (0%)
	Some high school/GCSE level (in UK)	45 (6.88%)	43 (5.52%)	1 (3.33%)	0 (0%)
	High school diploma/A-level education (in UK)	97 (14.83%)	52 (6.68%)	1 (3.33%)	3 (10.71%)
	Some university (or equivalent)	179 (27.37%)	232 (29.78%)	4 (13.33%)	8 (28.57%)
	Bachelor's degree (or equivalent)	193 (29.51%)	261 (33.50%)	15 (50%)	9 (32.14%)
	Post-graduate degree (e.g. masters or doctorate)	132 (20.18%)	187 (24.01%)	5 (16.67%)	8 (28.57%)
*Employment status*	Unemployed	53 (8.1%)	57 (7.32%)	7 (23.33%)	1 (3.57%)
	Student	256 (39.14%)	38 (4.88%)	2 (6.67%)	0 (0%)
	Part-time job	98 (14.98%)	124 (15.92%)	0 (0%)	3 (10.71%)
	Full-time job	237 (36.24%)	472 (60.59%)	20 (66.67%)	24 (85.71%)
	Retired	10 (1.53%)	88 (11.30%)	1 (3.33%)	0 (0%)
*Nationality*	US – United States	199 (30.43%)	376 (48.27%)	N/A	1 (3.57%)
	GB – United Kingdom	128 (19.57%)	134 (17.20%)	20 (66.67%)	21 (75.0%)
	Other	240 (36.7%)	269 (34.53%)	10 (33.33%)	6 (21.43%)
*Psychiatric history*	Never been diagnosed with a psychiatric illness	440 (67.28%)	507 (65.08%)	0 (0%)	28 (100%)
	Has been diagnosed with at least one psychiatric illness	214 (32.72%)	272 (34.91%)	30 (100%)	0 (0%)
*Previous psychedelic drug use*	Never	62 (9.48%)	343 (44.03%)	21 (70%)	28 (100%)
	Only once	40 (6.12%)	75 (9.63%)	3 (10%)	0 (0%)
	2–5 times	148 (22.63%)	158 (20.28%)	4 (13.33%)	0 (0%)
	6–10 times	106 (16.21%)	65 (8.34%)	0 (0%)	0 (0%)
	11–20 times	109 (16.67%)	66 (8.47%)	0 (0%)	0 (0%)
	21–50 times	110 (16.82%)	46 (5.91%)	2 (6.67%)	0 (0%)
	51–100 times	39 (5.96%)	13 (1.67%)	0 (0%)	0 (0%)
	More than 100 times	40 (6.12%)	13 (1.67%)	0 (0%)	0 (0%)

This table displays demographic data for each of the four datasets used in the study. Percentages within parentheses represent portion of total original dataset.

### Factor structure

A PCA was conducted on *cohort* data to confirm the previous structure attained and screen it for reduction. A Kaiser–Meyer–Olkin (KMO) test of sphericity returned a value of 0.80 on the cleaned cohort data (*N* = 319), indicating the data were suitable for a factor analysis. Our analysis suggests the same essential structure found in Haijen et al. (2018), with an initial Cronbach's alpha of 0.72 for the unreduced, 12-item scale. Scale reduction by the correlation matrix technique described in the Methods resulted in removal of three items: ‘I am preoccupied with my work and/or life duties’, ‘I have strong expectations for the upcoming experience’, and ‘The environment/setting feels good for my upcoming experience’ (see online Supplementary materials for correlations matrices used in reduction). To assess the internal consistency of the revised 9-item scale, a PCA was conducted using *ceremony* data (*N* = 735, KMO = 0.85). Reduction of the scale improved Cronbach's alpha to 0.83. The results of the factor analysis yielded three sufficiently orthogonal factors, namely (1) *set* (Cronbach's alpha: 0.81) – on which items inquiring about a felt readiness for and openness to the experience loaded; (2) a factor we called *rapport* (Cronbach's alpha: 0.76) – on which items sampling feelings of interpersonal trust and environmental comfort loaded; and (3) an *intention* factor that included items sampling expectations and intentions (Table 1).

### Convergent validity

Convergent validity was assessed through correlation of IPPS scores with the *surrender* scale (Russ et al., 2019a; 2019b). Total IPPS scores significantly correlated with *surrender* (*r* = 0.455, *p* < 0.0001), with *set* showing moderate correlative strength (*r* = 0.433, *p* < 0.0001).

### Predictive validity

Used as a total score or factor scores, the IPPS values were significantly predictive of important outcomes in each of the naturalistic datasets (Table 3). In both, *set* negatively correlated with *challenging experience* (*r* = −0.303 and −0.251 for *cohort* and *ceremony* respectively) and in ceremony settings *rapport* positively correlated with EBI scores (*r* = 0.136).

**Table 3.** Correlations between IPPS scores and relevant acute outcomes

	MEQ	CEQ	EBI	Communitas
Cohort				
Total	0.098	**−0.383**	0.011	N/A
Set	**0.168**	**−0.303**	0.012	N/A
Rapport	−0.020	**−0.213**	−0.052	N/A
Intention	**0.137**	**−0.165**	**0.189**	N/A
Ceremony				
Total	**0.205**	**−0.226**	**0.113**	**0.273**
Set	**0.183**	**−0.251**	0.066	**0.204**
Rapport	**0.144**	−0.050	**0.136**	**0.300**
Intention	**0.096**	**−0.083**	**0.097**	**0.180**

This table displays correlations between total and factor scores on IPPS and relevant acute outcomes. Bold numbers indicate significant (*p* < 0.05) correlations.

#### Train–test regression

IPPS explained 6.76% of the variance in CEQ scores (adjusted *R*^2^ = 0.063, *F* = 14.5, *p* < 0.0001), with *set* significantly predicting *challenging experience* (*β* = −0.062, *p* < 0.001). When predicting MEQ, IPPS explained 4.23% of the variance (adjusted *R*^2^ = 0.0376, *F* = 9.05, *p* < 0.0001). In particular, *set* significantly predicted *mystical-type experiences* (*β* = 3.17, *p* < 0.001). Variance in EBI was explained to a lesser extent, accounting for 2.42% of the variance (adjusted *R*^2^ = 0.02, *F* = 5.12, *p* = 0.0017) and was significantly predicted by *rapport* (*β* = −0.062, *p* = 0.005).

#### Controlled studies regression

To test whether the predictions discovered through multi-dataset correlation and regression analysis were present in controlled datasets, we used regression fitting on two controlled datasets to explore if CEQ, EBI, and MEQ could be predicted by the IPPS. A simple regression fit in *depression trial* showed *rapport* explained 8.65% of EBI variance (*β* = 3.48, *p* = 0.028). In *healthy volunteers*, a simple regression fit showed that *set* explained 16% of CEQ variance (*β* = −3.72, *p* = 0.043). Also in *healthy volunteers*, a multiple regression fit showed that *set* and *rapport* explained 40% of MEQ variance (*p* = 0.003) (*set*: *β* = 3.44, *p* = 0.088, *rapport*: *β* = 10.75, *p* = 0.074).

### Retrospective potential

Administration of the IPPS before and after the psychedelic experience in cohort data provided an opportunity to investigate retrospective validity. We found that prospective IPPS scores significantly correlated with retrospective IPPS scores. This was true for total scores (*r* = 0.658, *p* < 0.0001), as well as individual factors *set* (*r* = 0.683, *p* < 0.0001), *rapport* (*r* = 0.462, *p* < 0.0001), and *intention* (*r* = 0.637, *p* < 0.0001).

## Discussion

This study sought to examine and develop the construct validity of a new ‘pre-state’ subjective rating scale for predicting acute responses to psychedelic compounds. We call this scale the ‘Imperial Psychedelic Predictor Scale’, or IPPS. Through data reduction and assessments of internal, convergent, and predictive validity, a brief 9-item scale was derived that contains three orthogonal factors, namely, *set*, *rapport*, and *intention* that were differentially predictive of different aspects of acute psychedelic experience.

As hypothesized, a positive mindset, as indexed by *set*, encapsulating feelings of readiness, preparedness, and openness for the experience plus a sense of comfort, general good mood, and low anxiety was associated with higher scores on positive aspects of the acute experience, namely *mystical experience* and *emotional breakthrough* and lower scores on *challenging experience*. A generally consistent pattern was seen for *rapport* and *intention*.

The prediction of EBI by *rapport* as per previous work (Murphy et al., 2021) suggests that social relationships may play a key role in *emotional breakthrough*. Positive therapeutic relationships have been identified as an important component for effective therapy (Kamilar-Britt, Gordis, & Earleywine, 2023; Murphy et al., 2021), and ‘therapeutic alliance’ has been found to be a core common factor underlying therapeutic response to psychotherapy (Grencavage & Norcross, 1990; Tschacher, Haken, & Kyselo, 2015). Finally, acute and end-of-retreat *communitas* – which was correlated with *rapport* in this study – has been related to subsequent improvements in mental health outcomes (Kettner et al., 2021; Kettner et al., *preprint*; Watts et al., 2022). These data suggest that *rapport* may be a valuable 2-item measure of ‘positive therapeutic relationship’ that is predictive of positive outcomes.

The strength of the IPPS’ predictive capacity in the train–test regression analysis may appear modest compared with other literature (Davis et al., 2021; McAlpine et al., 2024; Roseman et al., 2019; Russ et al., 2019a, 2019b). However, we only removed participants if key data were absent, while other studies have selected subsets (Davis et al., 2021; Roseman et al., 2019) or performed other data cleaning (Russ et al., 2019a, 2019b). Studies have also utilized retrospective data for scale development (McAlpine et al., 2024; Russ et al., 2019a, 2019b). A hindsight bias could easily strengthen apparent predictive relationships in these models. McAlpine et al. (2024) were able to demonstrate the scale can perform prospectively, showing prospective prediction of long-term outcomes within a dataset from a psilocybin retreat. However, our IPPS remains the only prospectively developed predictor of acute outcomes, showing statistical significance across four independent datasets. Additionally, the bulk of the participants in the present study were in naturalistic settings, in which dose and setting were not controlled. Considering the large effect that dose and setting (Aday et al., 2021) can have on an experience, it is unsurprising that the effect of ‘pre-state’ variables is smaller in relative contribution. However, in scenarios that control for these components – and thus lessen the influence of uncontrolled confounds, we were able to detect stronger effects for the relationships between IPPS factors and acute outcomes, suggesting that the influence of ‘pre-state’ is stronger in controlled settings. Finally, brief scales have better response rates (Edwards et al., 2002), which is an extremely important consideration if the hope is that a scale be widely adopted and used e.g. as part of a registry of real-world data. This is especially important if the scale is to be used close in time to a ritualized procedure such as often occurs with psychedelic use or therapy, as it lessens the time commitment and burden psychological distraction.

Future work might seek to examine whether some personality traits interact with IPPS factors or converge with them to improve the ability to predict response. For example, prior work has identified trait ‘neuroticism’ as a predictor of *challenging experience* (Barrett, Johnson, & Griffiths, 2017), and other studies have identified additional factors including high doses, young age, current life stress, polydrug use – including mixing psychedelics with high potency cannabis, adverse environmental conditions, and poor rapport or absent supervision (Bremler et al., 2023; Carbonaro et al., 2016; Simonsson, Hendricks, Chambers, Osika, & Goldberg, 2023), and even female gender (Bienemann, Ruschel, Campos, Negreiros, & Mograbi, 2020), past diagnosis of personality disorder (Marrocu et al., *preprint*), and avoidant attachment style (Stauffer, Anderson, Ortigo, & Woolley, 2021) as predictors of adverse responses to psychedelics. Our new tool may aid in safeguarding, highlighting if and when use might be contraindicated and should be delayed, such that e.g. *set* and *rapport* can be worked on and improved. However, it is important to recognize that the simple predictors identified in the present work could only explain a modest proportion of the variance in response, the majority remaining unexplained. This highlights the need for more work on prediction of response modeling in this space. It also implies that it may be premature to apply the IPPS as a (trustworthy) safe-guarding tool.

### Limitations and future directions

This is a self-report study leaning primarily on correlation analyses. Previous efforts to predict long-term psychedelic outcomes have been mixed, so we focused on prediction of acute measures that could optimize safety and, after better models are developed, predict long-term therapeutically relevant outcomes as well. Future analyses such as structural equation modeling or path modeling may seek to identify causal structure in statistical relations to better predict key dependent variables such as mental health outcomes. Moreover, the influence of other core factors such as demographics, dose, setting, ‘matrix’ (Eisner, 1997), and ‘integration’ could be examined to see whether more powerful predictive models can be devised. In the meantime, the predictive power of the IPPS could be used to tailor treatment parameters, such as dosage or the amount or type of therapeutic support, with the knowledge that doing so can improve subsequent outcomes.

Another limitation pertains to how the scale was constructed i.e. based on a factor analysis on a relatively small pool of items generated by the senior author. More comprehensive approaches might include assessing online ‘trip’ reports, using interviews and surveys to inform item wording and to assess how items are understood across target populations, as well as the so-called Delphi technique, which involves utilizing the opinions of a carefully selected group of topic ‘experts’ (Sforzini et al., 2022). It is likely that we have overlooked salient factors in our choice of items for the IPPS. For example, none of our items explicitly measure *expectations* or *expectancy*. This construct could be treated either on a trait or state level. For example, in a recent double-blind randomized controlled trial of psilocybin therapy *v.* a conventional antidepressant for depression, *expectancy* was measured early in the trial as a single visual analogue scale item. Results showed that efficacy-related *expectancy* for psilocybin did not predict antidepressant response to psilocybin (Szigeti et al., 2024), yet the single-item approach worked very well for predicting response to a selective serotonin reuptake inhibitor – supporting its validity. Future research might consider assessing expectancy closer in time to drug in-take. In this respect, an expectancy item could be added to the IPPS.

Other limitations include the single item for the *intention* factor. This could be viewed as weak as there are no additional items with which to assess this factor's own internal validity. Accordingly, researchers may choose to omit this item to give an even more efficient scale (i.e. 8-items), or carry out further work to develop the construct validity of this specific factor. We also note that we have only one reverse scored item – i.e. ‘I feel anxious’ and thus, a similar critique could be made here.

Finally, we acknowledge that issuing ‘pre-state’ measures close to dosing could prime for certain responses and thus it is conceivable that this procedure could bias outcomes. One way this could be tackled in the future, would be to develop a scale with an equal number of items referring to positive and negative ‘pre-state’ conditions. In this regard, we acknowledge that e.g. with just one negative item in our present list i.e. ‘I feel anxious’, our scale could be accused of priming positive responses.

For scoring and use of the IPPS, as a first pass, we recommend using of *set* and *rapport* factors, independently, as predictors of acute or longer-term response (taking mean scores for each factor and excluding the single-item intention factor). Additionally, to be sensitive to negative priming, we recommend not including the single reverse score item of the ‘set’ sub-scale i.e. ‘I feel anxious’ prospectively. Thus, users of the IPPS should compute mean scores of the five positively scored items on the *set* factor and two items comprising the *rapport* factor, and test whether either (or both) is predictive of response.

### Summary

In summary, the IPPS is an intentionally concise rating scale that can be used to efficiently predict the quality of an acute psychedelic experience. The full 9-item scale assesses *set*, *rapport*, and *intentio*n, and a briefer 7-item, 2-factor (*set* and *rapport*) use of the scale is advised. Here we showed that the IPPS was predictive of *challenging* and *mystical-type experiences* as well as *emotional breakthrough*, all known to be predictive of subsequent mental health changes post psychedelic use. Our scale contributes to a growing knowledge base around *preparedness* that could inform the development of preparatory interventions to improve outcomes. Ultimately, we hope our development and open provision of the IPPS will serve to improve our collective ability to predict – and eventually improve responses to psychedelics. We see special value here given the increasingly liberalized use of these compounds and increased prevalence of use.

## Supporting information

Angyus et al. supplementary material 1 Angyus et al. supplementary material

Angyus et al. supplementary material 2 Angyus et al. supplementary material

Angyus et al. supplementary material 3 Angyus et al. supplementary material

## Supplementary material

The supplementary material for this article can be found at https://doi.org/10.1017/S0033291724002204.
