# Validation of the Imperial Psychedelic Predictor Scale – CORRIGENDUM

**DOI:** 10.1017/S0033291725101219

**Published:** 2025-08-26

**Authors:** Michael Angyus, Sarah Osborn, Eline Haijen, David Erritzoe, Joseph Peill, Taylor Lyons, Hannes Kettner, Robin Carhart-Harris

**Affiliations:** 1 Department of Brain Sciences, Centre for Psychedelic Research, Imperial College London, London, UK; 2 Department of Neuropsychology and Psychopharmacology, Faculty of Psychology and Neuroscience, Maastricht University, Maastricht, the Netherlands; 3 Departments of Neurology and Psychiatry, University of California San Francisco, San Francisco, CA, USA

When this article was originally published in Psychological Medicine it omitted a piece of supplementary material. This new addition is a scoring guide for the scale introduced in the paper.
