# Supplementary material for: Validation of the imperial psychedelic predictor scale
Source: Psychol Med. 2024 Sep 27;54(12):3539–47. doi: 10.1017/S0033291724002204 (PMC11496213; doi:10.1017/S0033291724002204)
Supplement: Angyus et al. supplementary material 1 — Angyus et al. supplementary material [file S0033291724002204sup001.docx]

**Table S1:** Correlations between Haijen’s IPPS items and relevant outcomes in the Cohort dataset.

| **Items** | **MEQ** | **CEQ** | **EBI** |
| --- | --- | --- | --- |
| I feel ready to surrender to whatever will be | **0.20** | **-0.13** | **0.12** |
| I am preoccupied with my work and or life duties | 0.03 | 0.08 | 0.05 |
| I feel open to the upcoming experience | **0.20** | **-0.16** | **0.11** |
| I feel well prepared for the upcoming experience | **0.16** | **-0.24** | 0.01 |
| I have a clear intention for the upcoming experience | **0.14** | **-0.17** | **0.19** |
| I feel comfortable about the upcoming experience | 0.09 | **-0.25** | 0.01 |
| I have strong expectations for the upcoming experience | **0.25** | 0.00 | **0.15** |
| I am in a good mood | **0.20** | **-0.19** | -0.03 |
| I feel anxious | 0.04 | **0.26** | 0.09 |
| The environment setting feels good for my upcoming experience | **0.22** | -0.09 | **0.12** |
| I have a good feeling about my relationship with the group people who will be with me during my experience | 0.04 | **-0.16** | -0.02 |
| I have a good relationship with the main person people who will look after me during the upcoming experience | -0.04 | **-0.18** | -0.06 |
| Table displays correlations between original items and relevant acute outcomes. Bold numbers indicate significant correlations (p<0.05). | | | |
