# Supplementary material for: Validation of the imperial psychedelic predictor scale
Source: Psychol Med. 2024 Sep 27;54(12):3539–47. doi: 10.1017/S0033291724002204 (PMC11496213; doi:10.1017/S0033291724002204)
Supplement: Angyus et al. supplementary material 3 — Angyus et al. supplementary material [file S0033291724002204sup003.docx]

**Table S3:** Correlations Significant in Both Datasets

| **Items** | **MEQ** | **CEQ** | **EBI** |
| --- | --- | --- | --- |
| I feel ready to surrender to whatever will be | X | X | X |
| I am preoccupied with my work and or life duties |  |  |  |
| I feel open to the upcoming experience | X | X |  |
| I feel well prepared for the upcoming experience | X | X |  |
| I have a clear intention for the upcoming experience | X | X | X |
| I feel comfortable about the upcoming experience |  | X |  |
| I have strong expectations for the upcoming experience |  |  |  |
| I am in a good mood | X | X |  |
| I feel anxious |  | X |  |
| The environment setting feels good for my upcoming experience | X |  |  |
| I have a good feeling about my relationship with the group/people who will be with me during my experience |  |  | X |
| I have a good relationship with the main person/people who will look after me during the upcoming experience |  |  | X |
| Table displays which items were repeatedly significantly correlated (p<0.05) with relevant outcomes. An ‘X’ indicates that the item was significantly correlated with the acute outcome in both the Cohort dataset and the Ceremony dataset. | | | |
